# A comparison of two treatments for childhood apraxia of speech: methods and treatment protocol for a parallel group randomised control trial

**DOI:** 10.1186/1471-2431-12-112

**Published:** 2012-08-03

**Authors:** Elizabeth Murray, Patricia McCabe, Kirrie J Ballard

**Affiliations:** 1 Speech Pathology, Faculty of Health Sciences, The University of Sydney, PO Box 170, Lidcombe, 1825, Sydney, Australia

**Keywords:** Childhood apraxia of speech, Treatment, Effectiveness, Randomised control trial, Intervention, Rapid syllable transition treatment, Nuffield dyspraxia programme

## Abstract

**Background:**

Childhood Apraxia of Speech is an impairment of speech motor planning that manifests as difficulty producing the sounds (articulation) and melody (prosody) of speech. These difficulties may persist through life and are detrimental to academic, social, and vocational development. A number of published single subject and case series studies of speech treatments are available. There are currently no randomised control trials or other well designed group trials available to guide clinical practice.

**Methods/Design:**

A parallel group, fixed size randomised control trial will be conducted in Sydney, Australia to determine the efficacy of two treatments for Childhood Apraxia of Speech: 1) Rapid Syllable Transition Treatment and the 2) Nuffield Dyspraxia Programme – Third edition. Eligible children will be English speaking, aged 4–12 years with a diagnosis of suspected CAS, normal or adjusted hearing and vision, and no comprehension difficulties or other developmental diagnoses. At least 20 children will be randomised to receive one of the two treatments in parallel. Treatments will be delivered by trained and supervised speech pathology clinicians using operationalised manuals. Treatment will be administered in 1-hour sessions, 4 times per week for 3 weeks. The primary outcomes are speech sound and prosodic accuracy on a customised 292 item probe and the Diagnostic Evaluation of Articulation and Phonology inconsistency subtest administered prior to treatment and 1 week, 1 month and 4 months post-treatment. All post assessments will be completed by blinded assessors. Our hypotheses are: 1) treatment effects at 1 week post will be similar for both treatments, 2) maintenance of treatment effects at 1 and 4 months post will be greater for Rapid Syllable Transition Treatment than Nuffield Dyspraxia Programme treatment, and 3) generalisation of treatment effects to untrained related speech behaviours will be greater for Rapid Syllable Transition Treatment than Nuffield Dyspraxia Programme treatment. This protocol was approved by the Human Research Ethics Committee, University of Sydney (#12924).

**Discussion:**

This will be the first randomised control trial to test treatment for CAS. It will be valuable for clinical decision-making and providing evidence-based services for children with CAS.

**Trial Registration:**

Australian New Zealand Clinical Trials Registry: ACTRN12612000744853

## Background

To date the consensus in the literature is that Childhood Apraxia of Speech (CAS) is a disorder of speech motor programming and planning with genetic, neurologic, or idiopathic causes [1-4]. Children with the disorder have significant speech impairments due to an inability to control placement and timing of lip, tongue and vocal movements. These impairments result in inconsistent productions of the same words, difficulty sequencing speech sounds together to form fluent words and sentences and impairments of the melody (i.e. prosody) of speech. The speech of these children is often unintelligible and the disability can persist throughout the lifespan, despite normal intelligence and comprehension of language [5,6]. The impaired development of speech has flow on effects, frequently disrupting development of reading, spelling and writing skills (e.g. learning letter-sound relationships for deciphering new words), social communication, and academic potential [6,7]. Adults with speech disorders (including persistent CAS) are known to have a lower socio-economic status than non-speech disordered peers, an increased incidence of depression and may select occupations that require little or no personal interaction despite normal intelligence [6,8,9]. Speech pathologists frequently report that children with CAS make slow progress in treatment and show deterioration or loss of skills once treatment stops [10,11]. It is unclear if this is due to the child’s impaired motor learning or to a lack of evidence to inform effective treatment [10,12,13]. Due to CAS’s characteristics and associated risk factors, the ultimate aim of treatment must be long term maintenance of learned skills and generalisation of treatment effects to improvement across untrained but related speech skills and speaking contexts.

There are few rigorous treatment studies published for CAS currently and no randomised control trials to date [1,14]. The best evidence available consists of single case experimental designs and case series for a range of different treatments across different age ranges with samples sizes no greater than twelve (e.g. [15]). Published speech-focussed treatments with some replicated evidence include the Rapid Syllable Transition Treatment (ReST) [12,16], Dynamic Temporal and Tactile Cueing (DTTC) [17,18] and the Nuffield Dyspraxia Programme - Third edition (NDP3) [19-21]. Replicated evidence also exists for pre-reading and spelling interventions [15,22,23] and alternative or augmentative communication methods [24-28] that work on communication skills. These methods are the best poised to be utilised clinically at present and also to be included in any controlled trial. Other published evidence includes studies on melodic-intonation therapy (speech melody) therapy [29] and speech rate control [30]. Recently, intervention studies have included combined approaches such as a combined stimulability and core vocabulary approach [31] and a combined melodic intonation therapy and touch-cue method [32]; the PROMPT approach [33] and technological interventions providing graphic displays of tongue movements during speech such as electropalatography [20,34]. Moreover there are many published commercial programmes for CAS, which have no published evidence to date. Examples include the Kaufman Speech Praxis Treatment Kits [35-37] and the Easy Does It For Apraxia series [38,39]. It is relevant to note that children with CAS have different needs as their skills change with maturation and therapy and so it is plausible that multiple treatments will be effective but for different symptoms, severities and ages. In summary, while consumers and clinicians have a range of potential intervention programmes available, few have been experimentally tested and none have been experimentally compared. A Cochrane review on the topic states “there is a critical lack of well controlled treatment studies addressing treatment efficacy for CAS” [14] pg 2.

### Nuffield Dyspraxia Programme – Third edition (NDP3)

At time of writing, the Nuffield Dyspraxia Programme is in its third edition and has been utilised in clinical practices in Australia and the United Kingdom for approximately 30 years [40,41]. The comprehensive treatment package includes its own assessment as well as a list of over 500 words to be targeted in therapy, and corresponding therapy stimulus pictures. The theoretical basis of the programme is that motor learning is complex and hierarchical; one needs to perform frequent and systematic practice to master foundation levels before progressing to harder, more complex speech patterns. Here mastery is defined as fluent, automatic and independent production of targeted speech behaviours. To date, a total of five empirical case studies have demonstrated positive outcomes for this treatment alone [19,21], or in conjunction with electropalatography therapy [20]. Given these results and the programme’s broad dissemination, testing the programme in a randomised control trial is appropriate.

### Rapid Syllable Transition Treatment (ReST)

The ReST treatment is a more recently conceived and tested treatment approach [12]. The programme is grounded in theory of motor control and learning and is informed by an extensive motor learning literature [42]. The ReST treatment applies Principles of Motor Learning [43] with an aim to maximise long-term maintenance and generalisation of treated speech skills in children with CAS. ReST involves intensive practice in producing multisyllabic pseudo-words (e.g. toobiger) to improve the accuracy of speech sounds, the ability to transition rapidly and fluently from one sound/syllable to the next, and control of the melody in the form of relative emphasis, or stress, placed on each syllable within a word. Pseudo-words are used to mimic novel word learning and this allows children to develop and practice new speech patterns without interference from existing erroneous speech patterns. There is published evidence that the effects of speech treatment using phonotactically permissible pseudo-words (non-words) generalise to improved production of real words of similar or easier composition and complexity [44]. ReST involves two components within each treatment session. The first is a Pre-Practice (or training) Component where the stimuli are taught with cues to shape accurate production and immediate, specific feedback is given after each production. This is followed by a longer Practice Component incorporating those Principles of Motor Learning that have been shown to facilitate long-term learning and generalisation of skill. For example, “right/wrong” (i.e. Knowledge of Results or KR) feedback is provided by the clinician, fading from high frequency to low frequency over the session (i.e. provided on a total 50% of responses) and this KR feedback is delayed with a silent interval between response and feedback to encourage self-evaluation. Two published case series involving a total of ten children [12,16] indicate strong clinical effects for ReST treatment, motivating further study in a phase three trial.

Based on these foundational studies, we are well poised to conduct a larger scale clinical trial of ReST treatment against NDP3. Our hypotheses for this planned randomised control trial are: 1) treatment gains from pre-treatment to 1 week post-treatment will be similar for both treatments, 2) ReST treatment will result in greater maintenance of treatment effects at 1 and 4 months post-treatment than NDP3 treatment, and 3) ReST treatment will result in greater generalisation of treatment effects to untrained but related speech behaviours at 1 week, 1 month and 4 months post-treatment than NDP3 treatment.

## Methods and design

A parallel group, fixed size randomised control trial will be conducted in Sydney, Australia to determine the comparative efficacy of two treatments for Childhood Apraxia of Speech: the Rapid Syllable Transition Treatment (ReST) and the Nuffield Dyspraxia Programme – Third edition (NDP3). This protocol has been approved by the Human Research Ethics Committee of the University of Sydney (approval number 12924).

A power calculation has been used to determine the number of participants to be recruited. Previous pre and post treatment data from within-subject ReST studies (e.g. [12,16]) for a total number of 14 participants demonstrated statistically significant differences for 12 of the 14 participants and large effect sizes (Cohen’s d = 1.36). Based on this, the estimated sample size to detect a reliable treatment effect (alpha level 0.05, beta level 80%) is 6 per group for this RCT. We have no estimates for the NDP3 due to lack of published effect sizes or data to enable calculation of an effect size. On this basis we propose 10 per group to allow for attrition and to accommodate the higher heterogeneity with a between-subjects design.

### Participants

Participants will be recruited via flyer and email advertisement and all interventions will be undertaken at the University of Sydney. The participant’s caregivers will be asked to provide informed consent for a child to participate prior to admission into the study in accordance to our ethics approval.

Inclusion criteria will be: a) clinical diagnosis of suspected CAS, b) between 4 and 12 years of age, c) comprehension skills within 1 SD of the mean for a child’s chronological age (based on normative data for the receptive language score from the Clinical Evaluation of Language Fundamentals (CELF) - Fourth edition or CELF Preschool - Second edition [45,46], d) normal or adjusted-to-normal hearing and vision, e) the child and at least one parent as native Australian English speakers, and f) no other diagnosed developmental/genetic disorders.

### Assessment process

#### Screening

All potential participants will be screened over the phone and by requesting any speech pathology or other relevant reports to determine if children are likely to meet the inclusion criteria. Any overt contraindications should be determined at this time (e.g. other developmental diagnoses).

#### Eligibility assessment

All potential participants who are not excluded during telephone screening will participate in a two-hour assessment to ensure they meet the inclusion criteria and meet the diagnostic criteria for CAS. The current gold standard in CAS diagnosis is expert clinical opinion as there are no validated standardised assessments available [13]. Accordingly CAS diagnosis will be made by the first author according to the ASHA (2007) criteria [1] as well as Strand’s 10 point checklist [47] where each of the ten items are assessed across three tasks and a diagnosis of CAS is made if a child scores four or higher on the checklist. Diagnoses will be verified by the second author blinded to the first author decision.

The eligibility assessment includes a case history questionnaire, hearing screening [48], the CELF [45,46] to determine age appropriate comprehension skills, completing three repetitions within the Inconsistency subtest of the Diagnostic Evaluation of Articulation and Phonology (DEAP) [49] to determine percentage within-word consistency across 25 words, the Test of Polysyllables [50,51] to document phonemic and phonetic level errors in words with greater complexity, a 100-utterance connected speech sample [52] and an oral structural and functional examination [53].

#### Diagnostic assessment

Eligible participants will undergo a further two hours of assessment to describe their broader communication skills. The further assessment will test memory and processing skills using the Children’s Test of Nonword Repetition [54], Comprehensive Test of Phonological Processing [55], Test of Auditory Processing Skills (3^rd^ edition) [56] (verbal memory, auditory discrimination); processing and production of prosody using the PEPS-C Test of Prosody [57]; and severity of articulation disorder using the Goldman-Fristoe Test of Articulation - Second edition (GFTA-2) [58]. Finally, an estimate of verbal cognitive ability will be obtained with the Peabody Picture Vocabulary Test – 4 [59,60] .

#### Baseline assessment probe

One further hour of assessment will be undertaken using a specially designed probe of 292 single sounds, words and phrases using a PowerPoint presentation to establish baseline performance on treated and untreated experimental stimuli. The first 162 stimuli are the Nuffield Dyspraxia Programme’s Dyspraxia Assessment stimuli [41] consisting of a variety of single consonants (e.g. ‘s’ or ‘d’), vowels (e.g ‘ah’ or ‘oo’), one syllable words (e.g. ‘no’, ‘up’ and ‘moon’), two syllable words (e.g. ‘dinner’ and pocket’), multisyllable words (e.g. ‘banana’ and ‘caterpillar’), words with clusters or blends (e.g. ‘**st**ar’ or ‘fa**st**’) and phrases (e.g. ‘do you like jelly?’ and ‘I saw five camels at the zoo’). In addition, 80 nonsense words will be probed as stimuli for the ReST program, with 40 designated as treatment stimuli and another 40 as untreated stimuli. Each set of 40 words will be individualized based on the child’s speech skills and stimulus selection criteria for the ReST programme. Twenty will be at the child’s current level (e.g. two syllable CVCV words e.g. ‘tegar’) and the other twenty will be the related next step (e.g. ‘tegarfer’) to allow for assessment of treatment gains if progression criteria are met in the intervention period. The final component of the probe will be 50 untreated words of one, two and three syllables to measure potential generalisation of treatment effects to untreated speech behaviours for both treatments. All real words will be pictured and written on screen. All ReST stimuli will be written on screen. Participants with adequate reading skills will read the pseudo-word stimuli, those who cannot read will be asked to imitate a model. A pre-recorded model of all stimuli said by one Australian English speaker will be available to ensure consistency in delivery across examiners if imitation is required. The PowerPoint presentation will also include built in standardised breaks and animations to promote interest and motivation for the participants. Each participant will have individualised stimuli chosen for them for both programmes based on their assessment performance prior to randomisation, group allocation and treatment.

### Group allocation

Participants will be consecutively allocated a participant number from 1–30. Participants will be randomised to treatment condition utilising concealed allocation. That is, cards with treatment name (ReST or NDP3) will be placed in opaque envelopes, an independent person will then randomly draw envelopes and consecutively place participant numbers 1 to 30 on the front of the envelopes without opening them. In this way, each eligible participant will be randomly allocated to treatment according to their assigned participant number.

### Intervention delivery and dosage

The two treatments will be conducted in parallel within three treatment blocks over school holiday time in Sydney, Australia, permitting a maximum of 10 participants per block to allow for recruitment of between 20 and 30 participants across 3 treatment blocks. Treatments will be delivered by trained and supervised speech pathology clinicians. Each child will be allocated one clinician for all treatment sessions. Clinicians will be blinded to hypotheses. The parents of children will not be informed of the name and strategies of the treatment their children are receiving until after data collection is completed to ensure no home practice will be attempted.

All participants, regardless of treatment condition, will receive the same amount of time in treatment with 1-hour sessions, four times a week for three weeks for a total of 12 treatment hours. All participants will complete between 100–120 production trials on treatment words per 1-hour session (i.e. a total of 1200 to 1440 responses during treatment), to match the practice intensity and session distribution of our pilot studies of ReST [12,16] and the current best evidence of treatment intensity for CAS [61]. For the NDP3 programme this will equate to approximately 30–40 trials during each 18 minute period for each goal. For the ReST programme, this will equate to 5–20 Pre-Practice trials and 100 practice trials.

#### Nuffield Dyspraxia Programme – Third edition (NDP3)

We have operationalised the NDP3 programme with the assistance of its original authors to ensure dosage and other aspects will be controlled with the other treatment and yet we will be faithful to the original programme. Therapy will be conducted in accordance with the tenet of the NDP3 and the operationalised manual which was developed for this study, and treatment fidelity will be evaluated to ensure compliance.

##### Treatment goals and stimuli

In the NDP3, treatment goals are selected through assessment of a child’s speech sound, melody (prosody), vocal and nasal quality strengths and needs. Three independent goals will be selected according to the NDP3 hierarchy ‘Treatment Planning and Progression’ checklist in the original manual [41] with the intention of building on their strengths to facilitate learning of a new speech pattern or incorporate an old pattern or sound into a new more complex context (e.g. a two rather than one syllable word). Each of the three individualised goals will involve the child naming five different pictured stimuli independently with 90% accuracy. Cues are provided as needed but only independent productions over at least 12 opportunities are counted towards achieving the goal.

Five sounds or real word stimuli will be chosen per goal (for example if the goal is ‘single consonant sounds’, initially selected stimuli will include sounds the child can produce at least 10%–60% of the time with cues but would benefit from therapy (e.g. ‘s’, ‘h’ ,‘f’, ‘m’ and ‘y’). The original NDP3 pictured stimuli will be used in sessions [41]. Non-speech (oral-motor) goals will be excluded from this study to ensure consistent dosage of speech production intervention across treatments within each treatment session.

##### Treatment procedure

Each of the three goals will be worked on in a block of 18 minutes during every session using game activities. Each treatment task will incorporate verbal instructions, modelling, articulation, visual-tactile cues to guide tongue/lip placement (e.g. cued articulation [62]) and pictured stimuli to guide children in modifying their production to say the sound or selected stimuli accurately. Immediate feedback describing the child’s performance (e.g. ‘You made a great ‘s’ sound with your teeth together!’) will be given after every production attempt (i.e. on 100% of responses). When a production is accurate, the child will be asked to repeat the stimulus item stimuli three times to help consolidate new motor plans. The child will need to say each stimulus item with 90% accuracy out of at least 12 trials without modelling or cueing from the clinician before it is removed from treatment and a new target word is introduced. Production accuracy for the acquired word(s) will be probed at the start of each goal every second session to ensure maintenance of skill. If performance deteriorates, it will be re-introduced into the set of treatment words and the newest item removed to maintain the same number of treatment words at all times. Once 5 stimulus items are acquired for a given goal, treatment will move up the hierarchy to the next level (e.g. once a consonant-vowel (CV) syllable is achieved, consonant-vowel-consonant-vowel (CVCV) sequences using the same speech sounds will be targeted).

##### Data collection

Accuracy of productions will be scored in every session to track percent accuracy for each stimulus item and the number of production attempts requiring clinician cues. These measures will be used to track treatment gains and in determining if and when to change treatment targets and/or levels according to the operationalised NDP3 instructions for this study.

#### Rapid Syllable Transition Treatment (ReST)

The ReST treatment was initially manualised by the second and third author from a verbal description of ReST treatment by its developer Professor Donald A. Robin, and with assistance from Jeannie McDonald. The first author adapted the stimuli and further operationalised the treatment to ensure experimental control in the context of this parallel RCT. The adapted manual will be used.

##### Treatment goals and stimuli

Treatment for all children will involve correctly producing selected pseudo-word stimuli. Each treatment session has two components, each with a different goal. In the Pre-Practice component, the goal is to elicit a total of five correct productions of any of the pseudo-word stimuli from the participant. Once this is achieved, treatment moves to the Practice component, where the goal is for the participant to produce the 20 pseudo-word stimuli 5 times each (n = 100), in random order, with 80% accuracy.

The pseudo-word stimuli for the programme are designed to be read and conform to phonotactic rules of real English words [63,64]. Pseudo-words will be either at a two syllable (CVCV where C = consonant and V = vowel sound) or 3 syllable (CVCVCV) level to target the level immediately above each participant’s speech skills at assessment. Twenty child-specific pseudo-words/phrases will be constructed and used for all 12 treatment sessions: 10 will have a weak-strong prosodic pattern (e.g. ‘tebor’ or ‘teborfer’) and 10 will have a strong-weak stress (e.g. ‘farbe’ or ‘farbegee’). The consonant (C) sounds in each word are individualised for each participant to ensure a) they are sounds they can produce at least 10% of the time, b) they are maximally different in terms of place and manner of articulation and voicing to increase variation and complexity and c) are not vowel-like consonants (e.g. ‘y’ or ‘l’), to facilitate later acoustic analysis. Examples of consonants which may be included are: ‘f,’ ‘b’, ‘g’, and ‘t’, ‘m’ and ‘n.’ Vowel sounds will remain consistent across participants to ensure the stress patterns remain. No double-vowels or diphthongs (e.g. ‘eye’ or ‘ay’) will be included.

##### Treatment procedure

Each session will start with the Pre-Practice component where the child’s productions of the pseudo-words are shaped so s/he experiences correct responses. For the first two sessions, the pseudo-words will be randomly presented to gain an idea of areas of difficulty for each participant. From session three to twelve, difficult pseudo-words that require more shaping by the clinician will be presented as a primary focus to ensure correct productions are experienced in Pre-Practice. Cues will include all, or a subset of, modelling, tapping and breaking words word up into parts and then putting them together again. Knowledge of performance (KP) feedback will be given for all trials (e.g. for the prosodic pattern: “Great soft then strong beat, well done” or “you said all strong beats.”). The Pre-Practice component will take most of the session in the first two sessions, but for the majority of sessions will take approximately the first 10 minutes. Once any five pseudo-words are produced correctly, the treatment session will move into the Practice component.

In the Practice component, the 20 pseudo-words will be presented orthographically (in written form), in random order for a maximum of 100 production attempts by the child. No cues will be provided. Knowledge of results (KR), or “right/wrong”, feedback will be given after a three second delay for 50% of the 100 trials based on a decreasing feedback schedule (e.g. a randomly selected 9/10 given feedback for the first ten stimuli down to 1/10 for the final ten stimuli). After each set of 20 trials, participants will be given a timed 2 minute break (e.g. to play games or be active) to facilitate attention and compliance. Once a child produces ≥80 of the 100 productions correctly in the Practice component in two consecutive sessions, therapy progresses to the next level of difficulty. For children who started with 2 syllable pseudo-words (e.g. ‘tebor’) they will move on to the related 3 syllable pseudo-words (e.g. teborfer’). For children who started with 3 syllable words they will move on to putting the 3 syllable words into carrier phrases (e.g. ‘Can I have a begarter?’ or ‘It’s his fargeber’). The Practice component will comprise the majority of each session (approximately 50 minutes) after sessions 1 and 2.

##### Data collection

In the Pre-Practice component, total correct productions will be judged perceptually. Each pseudo-word will be counted for speech sound accuracy and for production of prosodic pattern (i.e. syllable stress) and for fluency in sequencing/transitioning of sounds within the pseudo-word. This will ensure that each participant is moved to Practice only after producing five correct responses and allow identification of areas that need more shaping in Pre-Practice. In Practice, data sheets will list all 100 pseudo-words in randomised order with asterisks identifying which items are to receive KR feedback. Space will be provided for the clinician to take data on whether the production of the whole pseudo-word was judged correct and, if any aspect was incorrect, the specific sub-parameter(s) in error: speech sounds and/or prosody and/or sequencing control.

### Treatment fidelity

Treatment fidelity, that is inter-rater reliability for (a) accuracy of response transcription and (b) application of the manualised protocol in terms of provision of cues, feedback and repetitions by the clinician, will be calculated between the clinician and the first author for 10% of each treatment session. This will document whether agreement for both measures is ≥85% (the benchmark for this study). If any variations occur for any treatment session, the first author will discuss this with the clinician contemporaneously to resolve disagreements in protocol application for future sessions.

### Outcome assessment

#### Outcome assessments and measures

Two hour post-treatment assessments will be scheduled for 1 week post treatment, 1 month post-treatment (after the no-treatment period of 4 weeks) and 4 months post-treatment (after resuming the participant’s usual speech pathology services for up to 3 months). All assessments will be completed by blinded independent assessors, that is no assessor will see the same child twice. Intra-rater and inter-rater reliability will be calculated for 10% of every probe completed to ensure transcription and judgments are above 85%. If the benchmark is not met, assessors will need to re-transcribe the recordings before discussion on items of disagreement to reach consensus.

The primary outcome will be speech (articulation) and prosodic (speech melody) accuracy on the specifically designed 292 item probe [49] administered during baseline and the three post-treatment assessments. Secondary outcome measures will include a) change in percent inconsistency across three repetitions of twenty-five words on the DEAP Inconsistency subtest and b) percent phonemes correct on the Test of Polysyllables and GFTA - 2 administered only at 1 month post-treatment. The probe and all assessments will be phonetically transcribed using the International Phonetic Alphabet, except the DEAP Inconsistency subtest which will use transcription methods according to the test manual.

#### Data analysis

Clinical effect sizes will be reported using Cohen’s d. A series of repeated measures ANOVA with between subject factor of treatment (NDP3, ReST) and within subject factor of time (pre-, 1 week post-, 1 month post-, and 4 months post-treatment) will be used to determine the relative efficacy of the two treatments as measured by the 292-item experimental probe. Separate analyses will be run for treated stimuli, to document treatment and maintenance effects, and untreated stimuli, to document generalisation of treatment effects and their maintenance. Additional repeated measures ANOVA (2 groups x 4 time points) will be run for the DEAP Inconsistency subtest [49], GFTA-2 [58] and the Test of Polysyllables [50,51]. If there is any attrition, an intention-to-treat analysis will be conducted.

### Recording and reliability

All assessment and treatment sessions will be audio-recorded using a Sony ICD-UX71F recorder, and video-recorded with a Cinde 88 audio-visual-system. The first two authors will supervise speech pathology clinicians using the in-house closed circuit video system to observe sessions from a different room in real-time with minimal distraction for the children. Professional recorders (Echo Layla 24/96 multitrack recording system, Marantz PMD660 solid-state recorder and a Roland Quad-Capture UA-55) will be used for high-quality audio-recording of children’s speech for all experimental probes and assessments. Recordings will use 16Bit and 48,000 Hz sampling rate to allow for future acoustic analysis, if warranted.

## Discussion

### Potential significance

This will be the first randomised control trial to test any treatment for CAS [14]. It is predicted that this will be valuable for clinical decision making and ultimately for providing evidence-based and timely services for children with CAS which may improve access to services and reduce the burden of the disorder. Potentially both treatments will be shown efficacious to some degree or for specific behaviours, which offers two evidence-based options tested at a gold standard level. If the treatments are successful, further work in ensuring time-efficient translation into practice can be pursued in effectiveness studies, knowing the treatments are efficacious and such work is warranted. Examples of this may include computer-based delivery of treatment to promote home practice and achieve treatment intensity with fewer face-to-face clinician-directed sessions.

As both treatments also vary in their application of Principles of Motor Learning, future investigations of the treatments may explore the effects of these differences on long-term outcomes. Recently Maas and colleagues [13] used DTTC with varied frequency feedback in treatment – low frequency (feedback on 60% of trials) and high frequency (feedback on 100% of trials). Two participants responded more favourably to low frequency feedback yet another responded more favourably to high frequency feedback, and regardless of feedback frequency generalisation was limited. Due to a sample size of three, it is unclear why these patterns may have occurred. Additionally Maas and colleagues [65] explored blocked versus random practice in terms of long-term maintenance and transfer. Again the results were mixed with two participants favouring blocked practice, another performing better with random practice and the fourth participant responding to neither condition. Our research will not explicitly test these motor learning principles, as we are using the treatments as faithfully as possible to their original form. However, the two treatment approaches differ in their application of key principles: the NDP3 uses high frequency feedback while ReST uses high frequency feedback in Pre-Practice and low frequency feedback in practice; the NDP3 uses blocked practice on specific speech targets but random presentation of words within blocks while ReST uses random practice in the Practice component. It will therefore be interesting to analyse the results for each treatment considering the inherent Principles of Motor Learning utilised.

### Potential limitations

As this is a behavioural treatment, it is not possible to blind clinicians and participants to treatments as per CONSORT [66] and PEDro guidelines [67]. Even though parents will not be told the name or details of the treatment their child will receive until their participation has ended, they could still talk to another parent in the waiting room and realise the treatments are different.

Clinicians will conduct all treatment sessions with two assigned participants using the ReST treatment for one and the NDP3 treatment for the other. Thus they will be required to be trained in and deliver both treatments. This is to reduce potential clinician effects. There is potential for drift across methods, motivating supervision of sessions by the authors and calculation of treatment fidelity on a portion of all treatment sessions.

## Abbreviations

CAS, Childhood Apraxia of Speech; NDP3, Nuffield Dyspraxia Programme – Third edition; ReST, Rapid Syllable Transition Treatment; DTTC, Dynamic Temporal and Tactile Cueing; CELF, Clinical Evaluation of Language Fundamentals; GFTA-2, Goldman Fristoe test of Articulation - Second edition; KP, Knowledge of Performance feedback; KR, Knowledge of Results feedback.

## Competing interests

The author(s) declare that they have no competing interests.

## Authors’ contributions

This manuscript was drafted by EM with PM and KJB contributing substantially to revisions. The ReST programme was developed by Professor Donald A. Robin and manualised by PM and KJB for Phase I and II studies. The ReST manual and stimuli were revised by EM for this study. Operationalised procedure for NDP3 was developed by EM with assistance from PM and KJB. Study design was developed using CONSORT and PEDro guidelines by all three authors. EM will carry out eligibility and baseline assessments; training and supervision of students conducting treatment will be completed by EM and PM; collection of data including post assessments, intra-rater and inter-rater reliability and treatment fidelity will be completed by EM. Data analysis will be completed by EM with guidance from PM and KJB and a statistician. All authors approve of the final version of this manuscript.

## Pre-publication history

The pre-publication history for this paper can be accessed here:

http://www.biomedcentral.com/1471-2431/12/112/prepub
